# Special Issue “New Frontiers in Facial Surgery”

**DOI:** 10.3390/jcm11113082

**Published:** 2022-05-30

**Authors:** Giovanni Dell’Aversana Orabona, Fabio Maglitto, Vincenzo Abbate, Antonio Romano, Paola Bonavolontà, Luigi Califano

**Affiliations:** Head and Neck Section, Department of Neurosciences, Reproductive and Odontostomatological Science, Federico II University of Naples, 80138 Naples, Italy; fabio.maglitto@unina.it (F.M.); vincenzo.abbate@unina.it (V.A.); romano.antonio1972@gmail.com (A.R.); paola.bonavolonta@unina.it (P.B.); bincenzo1@hotmail.it (L.C.)

Facial surgery remains a challenge for surgeons in order to achieve the best results, both functionally and aesthetically.

In recent decades, different new technologies have been developed to improve the predictability of results, such as Virtual Surgical Planning (VPS) [1].

Due to the complexity of the facial anatomy in the three-dimensional plane, VPS represents an important step forward in order to obtain more standardized and predictable results. VPS is the first step of Computer-Assisted Surgery (CAS), and its first application in cranio-facial surgery can be traced back to the 1980s [2].

Rising from the data acquisition of CT or MRI imaging, 3D analysis, surgical simulation, using CAD/CAM software, a 3D printed model is produced. This model can guide surgeons seeking to standardize the procedure, avoiding the surgeon-dependent variable. Different fields of application of this technology are reported in the literature, such as reconstructive surgery, traumatology, and orthognathic surgery [3].

The “New Frontiers in Facial Surgery” are also represented by new techniques developed in the field of facial cosmetic procedures.

When we discuss facial cosmetic procedures, we must focus on the most common, such as blepharoplasty, otoplasty, rhytidectomy (facelift), browlift, genioplasty and fat graft. Nowadays, minimally invasive procedures, such as the use of injectable filler and neurotoxin, also represent an actuality.

In particular, the development of piezosurgery radically changed daily practice in facial surgery. Piezoelectric tools were first applied in bone surgery. Different oral and dental procedures, such as third molar extraction, cyst excision, maxillary sinus lift, and implant surgery, were safety achieved using piezo surgery. The soft tissue sparing, in particular, of nerves and vessels made piezosurgery irreplaceable in the surgical armamentarium. Consequently, due to the possibility of performing osteotomies without neurovascular damage, this technology has found applications in craniofacial surgery. The possibility of using piezoelectric tools in rhinoplasty has increased in recent years [4]. The phase of osteotomies during rhinoplasty represents a delicate phase for the success of the procedure. Furthermore, osteotomies often result in pain, edema and bruising, all consequences that can be reduced thanks to the use of piezo. Reducing these postoperative sequelae increases patient satisfaction as well as the surgeon’s motivation to use new devices.

In 2007, Robiony et al. [5] reported the advantages of lateral osteotomies via the percutaneous approach using a piezo scalpel in their study. As reported by Keyhan et al. [6], the use of piezoelectric tools in rhinoplasty has favorable and valuable outcomes based on immediate postoperative morbidities.

As previously discussed, a current trend in facial surgery is to minimize the surgical approach. Surgical Endoscopy represents a valid tool to minimize the surgical approach to the facial skeleton. In the literature, different authors investigate the possibility of using a minimally invasive approach through endoscopic-assisted surgery. Abbate et al. [7] described the possibility of using a minimally invasive endoscopic approach to remove a midcheek mass. The proposed approach ensures the optimal visualization of the surgical field, although a longer learning curve could be considered the main disadvantage of this minimally invasive technique. Romano et al. [8] reported the endoscope-assisted enucleation of a mandibular odontogenic keratocyst. Through the endoscope-assisted approach, complete cyst removal is ensured, and the alveolar nerve is identified.

Current endoscopic application also includes facial traumatology, such as zygomaticomaxillary complex fractures, frontal sinus fractures, mandibular condyle fractures, orbital floor fracture, and medial orbital wall fractures [9].

As mentioned before, “New frontiers in facial surgery” are also represented by injectable filler and neurotoxin. Dermal filler and neurotoxin are useful in facial reshaping or facial wrinkles. These treatments allow patients who do not want to undergo surgery to obtain their desired facial correction with a good grade of tolerability.

In conclusion, this Special Issue aims to investigate the current technology in facial surgery, which represents an important field of surgery that can make lifesaving and life-changing transformations to patients’ individual lives and in society. Surgical techniques, computer-assisted surgery, biomaterials research, and minimally invasive nonsurgical and surgical procedures for facial rejuvenation and reconstruction warrant in-depth investigation in order to ensure better results for patients.
